# High-resolution DNA methylation screening of the major histocompatibility complex in multiple sclerosis

**DOI:** 10.3389/fneur.2023.1326738

**Published:** 2023-12-08

**Authors:** Qin Ma, Danillo G. Augusto, Gonzalo Montero-Martin, Stacy J. Caillier, Kazutoyo Osoegawa, Bruce A. C. Cree, Stephen L. Hauser, Alessandro Didonna, Jill A. Hollenbach, Paul J. Norman, Marcelo Fernandez-Vina, Jorge R. Oksenberg

**Affiliations:** ^1^Weill Institute for Neurosciences, Department of Neurology, University of California San Francisco, San Francisco, CA, United States; ^2^Department of Biological Sciences, University of North Carolina at Charlotte, Charlotte, NC, United States; ^3^Histocompatibility and Immunogenetics Laboratory, Stanford Blood Center, Palo Alto, CA, United States; ^4^Department of Pathology, Stanford University School of Medicine, Palo Alto, CA, United States; ^5^HLA Histocompatibility and Immunogenetics Laboratory, Vitalant, Phoenix, AZ, United States; ^6^Department of Anatomy and Cell Biology, Brody School of Medicine, East Carolina University, Greenville, NC, United States; ^7^Department of Biomedical Informatics and Department of Immunology and Microbiology, University of Colorado School of Medicine, Aurora, CO, United States

**Keywords:** DNA methylation, major histocompatibility complex, multiple sclerosis, differentially methylated regions, DNA methylation quantitative trait loci

## Abstract

**Background:**

The *HLA-DRB1* gene in the major histocompatibility complex (MHC) region in chromosome 6p21 is the strongest genetic factor identified as influencing multiple sclerosis (MS) susceptibility. DNA methylation changes associated with MS have been consistently detected at the MHC region. However, understanding the full scope of epigenetic regulations of the MHC remains incomplete, due in part to the limited coverage of this region by standard whole genome bisulfite sequencing or array-based methods.

**Methods:**

We developed and validated an MHC capture protocol coupled with bisulfite sequencing and conducted a comprehensive analysis of the MHC methylation landscape in blood samples from 147 treatment naïve MS study participants and 129 healthy controls.

**Results:**

We identified 132 differentially methylated region (DMRs) within MHC region associated with disease status. The DMRs overlapped with established MS risk loci. Integration of the MHC methylome with human leukocyte antigen (*HLA*) genetic data indicate that the methylation changes are significantly associated with *HLA* genotypes. Using DNA methylation quantitative trait loci (mQTL) mapping and the causal inference test (CIT), we identified 643 cis-mQTL-DMRs paired associations, including 71 DMRs possibly mediating causal relationships between 55 single nucleotide polymorphisms (SNPs) and MS risk.

**Results:**

The results describe MS-associated methylation changes in MHC region and highlight the association between HLA genotypes and methylation changes. Results from the mQTL and CIT analyses provide evidence linking MHC region variations, methylation changes, and disease risk for MS.

## Introduction

Multiple sclerosis (MS) is a chronic, immune-mediated disease of the central nervous system and common cause of neurological disability in young adults. A substantial body of epidemiologic and experimental research describes the heritable components of MS, including the identification of 233 independent genome-wide risk associations (1). The strongest association maps to the major histocompatibility complex (MHC) region (chr6:28,510,180-33,532,223), accounting in some models for up to 20% of the underlying disease susceptibility. The MHC is a remarkably gene-dense region of the human genome, and 32 out of the 233 independent MS risk associations were identified across the extended locus (1). The main susceptibility signal arises from the *HLA-DRB1* gene in the class II segment of the locus and HLA-DRB1*15:01 bearing haplotypes carry an average odds ratio risk of 3.08 that doubles for HLA-DRB1*15:01 homozygous genotypes (2). Additional HLA susceptibility alleles and haplotypes were identified as well as independent protective signals in the telomeric class I region of the locus (3–7).

HLA-encoded molecules are cell surface glycoproteins whose primary role in an immune response is to display and present short antigenic peptide fragments to T cells through specific receptors. Thus, antigen affinity and specificity determined by allelic sequence variation are considered the primary mediators of disease susceptibility. However, HLA expression levels are also important for T cell recognition, hence suggesting a role for epigenetic regulation, as an additional mediator of susceptibility in MS (8–10) and other autoimmune diseases (11–13). Among the different epigenetic signatures, DNA methylation is the best characterized epigenetic modification affecting gene regulation (14). However, due to its architectural complexity, standard whole genome bisulfite sequencing protocols or targeted arrays are inadequate to resolve the methylation landscape of the MHC locus with sufficient specificity and resolution. We utilized a set of validated capture probes to achieve high-throughput and high-resolution sequencing of the entire MHC and combined with bisulfite sequencing to conduct a comprehensive analysis of MHC methylation patterns in blood samples from treatment naïve MS participants and matched healthy controls.

## Materials and methods

### Subjects

Peripheral blood was sampled by venipuncture from 147 MS participants and 129 healthy controls recruited at the UCSF MS Center as part of the UCSF multiple sclerosis EPIC and ORIGINS studies (15). All MS participants were treatment naive (i.e., no glucocorticoids or disease-modifying MS therapy at any time prior to blood draw) and met clinical and radiographic diagnostic criteria for relapsing–remitting MS (RRMS). Eighty percent of cases were females, the mean age at sample collection was 43 (range from 18 to 65), and the mean Expanded Disability Status Scale (EDSS) was 1.5 (range from 0 to 6.5). Healthy controls were sex and age matched (mean age: 43, range: 24-61), and reported no history of autoimmune disease. All study participants were of white-western European ancestry. The study was approved by the UCSF Institutional Review Board, and informed consent was obtained from all subjects.

### Genomic DNA extraction and library preparation

Genomic DNA was extracted from peripheral blood using a standard salting-out method. An MHC-targeted bisulfite sequencing (BS-seq) approach was applied to generate base-resolution MHC methylome maps. Briefly, 500 ng genomic DNA was fragmented, end-repaired, A-tailed, and ligated to methylated adaptors. The ligated samples were pooled together to perform MHC capture using validated capture probes (16) and a custom targeted panel from Twist Bioscience following the target enrichment standard hybridization protocol (Twist Bioscience). The pooled fragments were then bisulfite converted using the EZ DNA Methylation Gold kit (Zymo Research) at 64°C for 2.5 h and amplified using the KAPA HiFi HotStart Uracil+ ReadyMix PCR Kit. Libraries were sequenced on HiSeq X 10 platform (Illumina). The sequencing reads were analyzed following an established bioinformatics pipeline. After filtering out low quality reads, the remaining paired reads were uniquely mapped to the reference genome (hg38, UCSC) using Bismark software with parameters: -N 1 -X 1000 --score_min L,0,-0.6. The bisulfite conversion rates were calculated using the lambda DNA. Subsequently, the 5mC level at each CpG site was computed as described. The number of “C” bases from the sequencing reads were counted as methylated (denoted as NC) and the number of “T” bases as unmodified (denoted as NT). The methylation levels were then estimated as NC/ (NC + NT). While previous array-based methods have less than 20% coverage on MHC region, our method can cover ~95% of the locus.

### HLA genotyping

DNA samples were processed for typing all alleles at the 11 classical HLA loci (HLA-A, -C, -B, -DRB3, -DRB4, -DRB5, -DRB1, -DQA1, -DQB1, -DPA1, and -DPB1) using MIA FORA NGS MFLEX HLA Typing Kits (Immucor Inc.) and sequenced using MiniSeq DNA sequencers (Illumina) as described (17). The MIA FORA 5.1 software with IPD-IMGT/HLA Database release version 3.44.0 was used for DNA sequence assembly and HLA genotype assignments.

### BS-seq data and statistical analyses

The metilene package (18) (version 0.2-8) was used to call the differentially methylated regions (DMRs), including only CpGs with at least 3× in coverage. DMRs were defined to have a 5% minimum absolute mean methylation difference, with a maximum distance of 1,000 nt between CpGs within a DMR and a minimum of 3 CpGs per DMR (parameter –M 1000 –m 3 –d 0.1). The Genomic Regions Enrichment of Annotations Tool (GREAT) (19) was employed to predict the DMR functional significance.

The correlations between HLA genotypes and methylation levels on DMRs were tested by ANOVA test in R. The differences in methylation levels between two groups were assessed with the Student’s t-test. The differences in the proportion of the HLA genotypes between two groups were assessed by Fisher’s exact test. Permutation test was performed to estimate the significance of the enrichment of DMRs in the ENCODE Encyclopedia Registry of candidate cis-Regulatory Elements (cCREs) (20). The BEDTools intersectBed function was used to get the overlap between the cCREs and DMRs. To test the statistical significance, the peaks were randomly permuted 1,000 times among human genome by BEDTools shuffleBed function, while retaining the length of each peak to assess the distribution of background overlap.

### Identification of DNA methylation quantitative trait loci

We used MatrixEQTL (21) to identify cis DNA methylation quantitative trait loci (cis-mQTL) in the ±500 kb genomic region flanking the DMRs. SNPs were pruned and selected using PLINK as those satisfying pairwise correlation *R*^2^ < 0.5 in a 250,000 bp window, with a window stride of 25,000 bp. Linear regression analysis was performed between the average methylation level of each DMR, and genotype encoded as 0, 1, or 2 copies of the reference allele in cis (±500 kb) of each DMR, accounting for the effects of age and sex. False discovery rate (FDR) was controlled follow the Benjamini–Hochberg procedure.

### Mediation analysis with causal inference test

The causal inference test (CIT) (22) was used to test for mediation of a known causal association between a single nucleotide polymorphism (SNP), and disease status putatively mediated by DNA methylation. Briefly, if we let S denote the SNP genotype, D denote the disease status, and M denote the potential mediator, DNA methylation, then the four component conditions are, (1) S and M are associated, (2) S and D are associated, (3) S is associated with M|D, and (4) S is independent of D|M. Test 4 requires an equivalence test and is implemented using a permutation based approach. A likelihood-based hypothesis testing approach is implemented for assessing causal mediation. The CIT was performed for the identified meQTL-DMR pairs using SNP information, DNA methylation level, and MS status from all subjects. One hundred permutations were performed to generate permutation-based FDR values to quantify uncertainty in the estimate. The permutations were specified the same for all tests to accurately account for dependencies among the tests. Permutation-based FDR values at or under 5% were used as cut off for significance.

## Results

### MS-associated DNA methylation patterns at the MHC region

**Figure 1:**
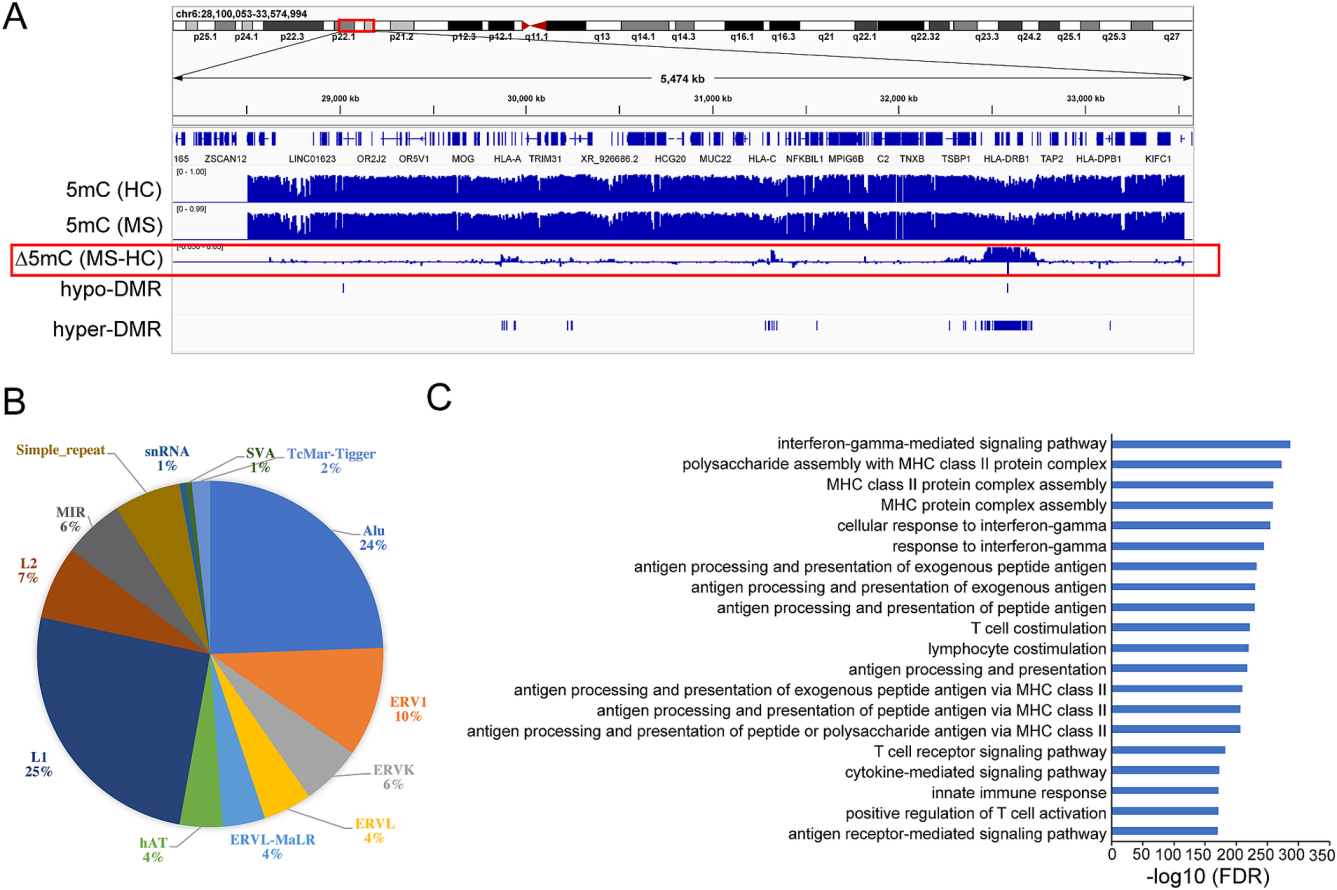
DNA methylation changes within MHC region in multiple sclerosis (MS) patients compared to healthy controls (HC). **(A)** Graphical representation of methylation (5mC) pattern within MHC region. ∆5mC highlighted in red box represents the methylation difference between MS and HC. **(B)** Pie diagram showing percentage of DMRs overlapping with different families of repetitive elements. **(C)** Functional annotation of the DMRs by GREAT. FDR: false discovery rate.

We generated base-resolution MHC (chr6: 28510180-33532223) methylome maps for 147 treatment naïve MS participants and 129 sex/age/ancestry matched healthy controls using targeted bisulfite sequencing. We then compared the methylome profiles between MS and controls to single out MS-associated methylation signatures, identifying 132 significant MHC DMRs (*p*-value <0.05, greater than 5% methylation difference): 3 DMRs were hypo-methylated and 129 DMRs were hyper-methylated compared to controls (Figure 1A) (Supplementary Table S1). Among the 132 DMRs, 41 hyper-DMRs and 1 hypo-DMR are significantly detected with FDR < 0.05 (Table 1), the latest covering exon 2 and extending into intron 1 of HLA-DRB1, predicted to function as a poised/active promoter and distal enhancer (Supplementary Figures S1A,B). Notably, 31 of the 32 100-kb regions around the established MS variants (1) overlap with the DMRs (Supplementary Table S2), whereas 81% concentrate within the HLA-class II region (Figure 1A) (Supplementary Table S1). One hundred and eight DMRs (82%) overlapped with repetitive elements (Supplementary Table S3). Most of them fall within the LINE-1 (L1) and Alu elements (25% and 24% of DMRs respectively) (Figure 1B). Seventeen DMRs coincide with or locate near non-HLA genes including *TSBP1*, *HCG24*, *HCP5B*, *LINC02571*, *LOC101929163*, *MIR6891*, *NFKBIL1*, *TRIM26*, and *ZNF311* (Supplementary Table S1). To gain additional insights into the regulatory function of the DMRs, we examined the enrichment in the ENCODE Encyclopedia Registry of candidate cis-Regulatory Elements (cCREs). The DMRs were significantly enriched in proximal enhancer-like signatures (fold change = 5.78, *p* = 0.002) and H3K4me3 peak regions (fold change = 3.94, *p* = 0.001). H3K4me3 is highly enriched at active promoters. The data indicate that DMRs are enriched in regulatory regions, especially in enhancer and promoter signals.

**Table 1:** MS-associated DMRs (FDR < 0.05) in blood samples.

Type	Chr	Start	End	Annotation	Overlap or nearby gene
Hypo-DMR	chr6	32,584,090	32,584,819	intron	HLA-DRB1
Hyper-DMR	chr6	30,240,007	30,240,096	intron	HLA-L
Hyper-DMR	chr6	32,346,304	32,346,315	intron	C6orf10
Hyper-DMR	chr6	32,501,153	32,501,446	Intergenic	HLA-DRB5, HLA-DRA
Hyper-DMR	chr6	32,510,757	32,511,014	Intergenic	HLA-DRB5, HLA-DRA
Hyper-DMR	chr6	32,514,164	32,514,539	Intergenic	HLA-DRB5, HLA-DRA
Hyper-DMR	chr6	32,524,049	32,524,870	intron	HLA-DRB5
Hyper-DMR	chr6	32,525,988	32,526,141	intron	HLA-DRB5
Hyper-DMR	chr6	32,528,094	32,528,550	intron	HLA-DRB5
Hyper-DMR	chr6	32,530,827	32,531,271	promoter-TSS	HLA-DRB5
Hyper-DMR	chr6	32,533,156	32,533,758	Intergenic	HLA-DRB5, HLA-DRB6
Hyper-DMR	chr6	32,534,320	32,534,555	Intergenic	HLA-DRB5, HLA-DRB6
Hyper-DMR	chr6	32,534,615	32,534,640	Intergenic	HLA-DRB5, HLA-DRB6
Hyper-DMR	chr6	32,535,314	32,535,799	Intergenic	HLA-DRB5, HLA-DRB6
Hyper-DMR	chr6	32,536,976	32,537,799	Intergenic	HLA-DRB5, HLA-DRB6
Hyper-DMR	chr6	32,539,703	32,539,829	Intergenic	HLA-DRB5, HLA-DRB6
Hyper-DMR	chr6	32,540,161	32,540,339	Intergenic	HLA-DRB5, HLA-DRB6
Hyper-DMR	chr6	32,543,751	32,544,442	Intergenic	HLA-DRB5, HLA-DRB6
Hyper-DMR	chr6	32,544,756	32,545,082	Intergenic	HLA-DRB5, HLA-DRB6
Hyper-DMR	chr6	32,545,600	32,546,249	Intergenic	HLA-DRB5, HLA-DRB6
Hyper-DMR	chr6	32,554,429	32,555,206	non-coding	HLA-DRB6
Hyper-DMR	chr6	32,557,405	32,557,611	intron	HLA-DRB6
Hyper-DMR	chr6	32,558,833	32,559,458	intron	HLA-DRB6
Hyper-DMR	chr6	32,563,339	32,565,354	Intergenic	HLA-DRB6, HLA-DRB1
Hyper-DMR	chr6	32,565,750	32,567,186	Intergenic	HLA-DRB6, HLA-DRB1
Hyper-DMR	chr6	32,571,487	32,572,114	Intergenic	HLA-DRB6, HLA-DRB1
Hyper-DMR	chr6	32,572,906	32,572,966	Intergenic	HLA-DRB6, HLA-DRB1
Hyper-DMR	chr6	32,574,091	32,575,342	Intergenic	HLA-DRB6, HLA-DRB1
Hyper-DMR	chr6	32,577,390	32,578,850	TTS	HLA-DRB1
Hyper-DMR	chr6	32,581,854	32,582,159	intron	HLA-DRB1
Hyper-DMR	chr6	32,582,710	32,583,620	intron	HLA-DRB1
Hyper-DMR	chr6	32,585,365	32,586,609	intron	HLA-DRB1
Hyper-DMR	chr6	32,587,104	32,587,856	intron	HLA-DRB1
Hyper-DMR	chr6	32,590,193	32,591,186	promoter-TSS	HLA-DRB1
Hyper-DMR	chr6	32,608,393	32,609,041	Intergenic	HLA-DRB1, HLA-DQA1
Hyper-DMR	chr6	32,621,746	32,622,065	Intergenic	HLA-DRB1, HLA-DQA1
Hyper-DMR	chr6	32,624,568	32,625,421	Intergenic	HLA-DRB1, HLA-DQA1
Hyper-DMR	chr6	32,639,100	32,640,436	intron	HLA-DQA1
Hyper-DMR	chr6	32,645,457	32,647,107	Intergenic	HLA-DQA1, HLA-DQB1
Hyper-DMR	chr6	32,662,687	32,663,415	intron	HLA-DQB1-AS1
Hyper-DMR	chr6	32,672,407	32,672,995	Intergenic	HLA-DQB1, HLA-DQB1-AS1
Hyper-DMR	chr6	32,686,397	32,687,030	Intergenic	HLA-DQB1, HLA-DQB1-AS1

The Genomic Regions Enrichment of Annotations Tool (GREAT) was applied to capture the biological functions associated with the DMRs (*p*-value <0.05). Gene ontology (GO) analysis on the genes overlapping with or located near DMRs indicated, unsurprisingly, an enrichment in immune related pathways, including interferon-gamma, MHC protein complex assembly, antigen processing, and presentation related pathways (Figure 1C).

### HLA genotypes are correlated with the methylation levels of DMRs

**Figure 2:**
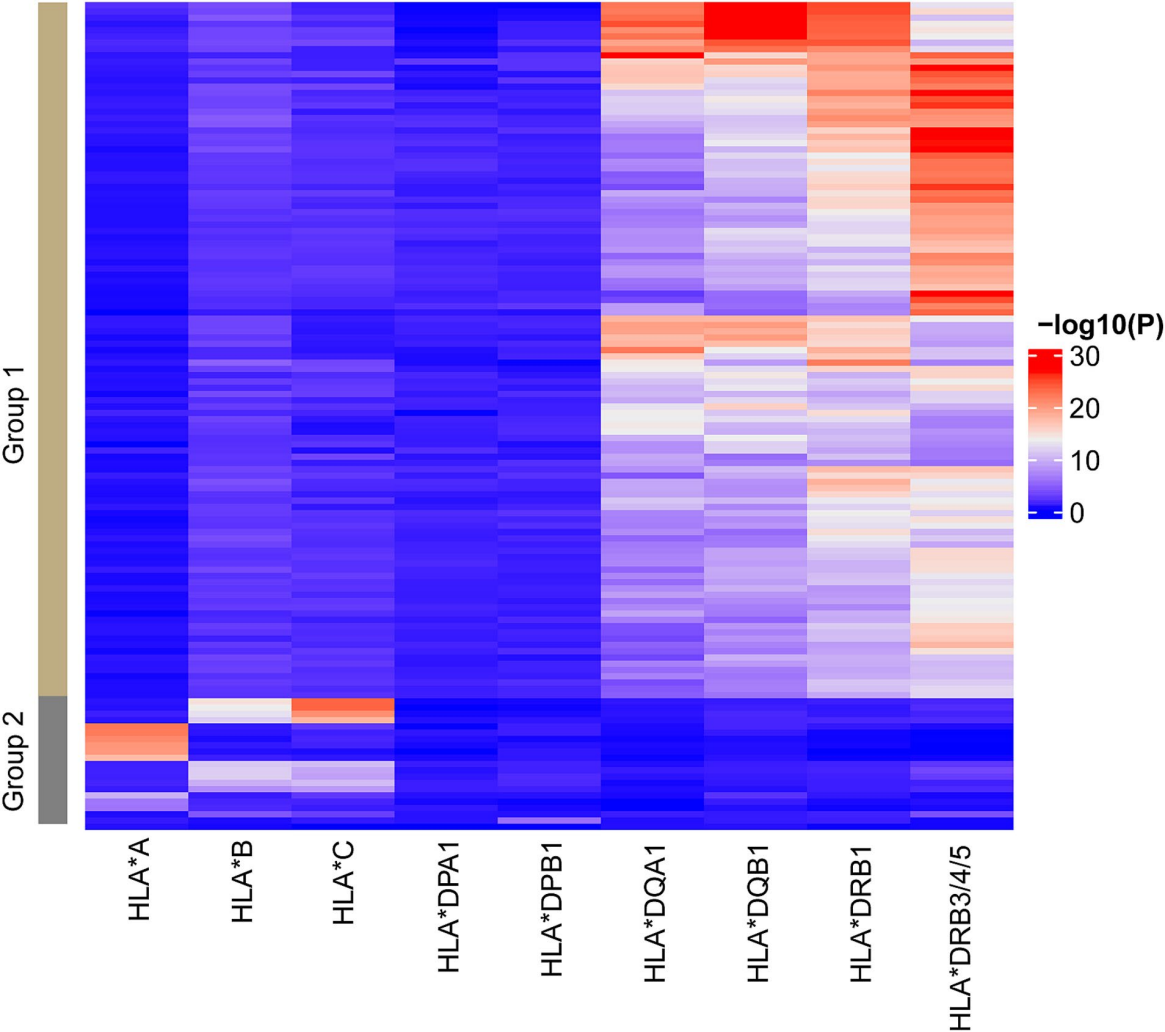
The corrections between HLA genotypes and DMRs. Heatmap representing the correlations between HLA genotypes clustered by classical HLA genes and the DMR methylation. The significances of the correlations are colored from blue to red to indicate low to high. The columns represent the HLA genotypes while the rows represent the DMRs. Two main groups of DMRs associated with HLA class I and class II genotypes, respectively, are labeled with group 1 and 2. Similar patterns were identified when separating cases and controls.

Given the concentration of DMRs in the HLA-class II region, we sought to address the association between *HLA* genotypes and MS-associated DNA methylation changes. All study participants were genotyped for the 11 classical HLA loci (HLA-A, -B, -C, -DRB3, -DRB4, -DRB5, -DRB1, -DQA1, -DQB1, -DPA1, and-DPB1), revealing significant associations between genotypes and DMR methylation levels (Figure 2 and Supplementary Table S4), specifically 9, 9, 1, and 112 DMRs (*p*-value <0.05) correlate HLA-A, HLA-B/C, HLA-DPB1 and HLA-DQA1/DQB1/DRB1/DRB3/4/5 genotypes, respectively (Figure 2 and Supplementary Table S4). DMRs clearly segregate into two main clusters, suggesting independent associations with class I and class II genotypes (Figure 2).

**Figure 3:**
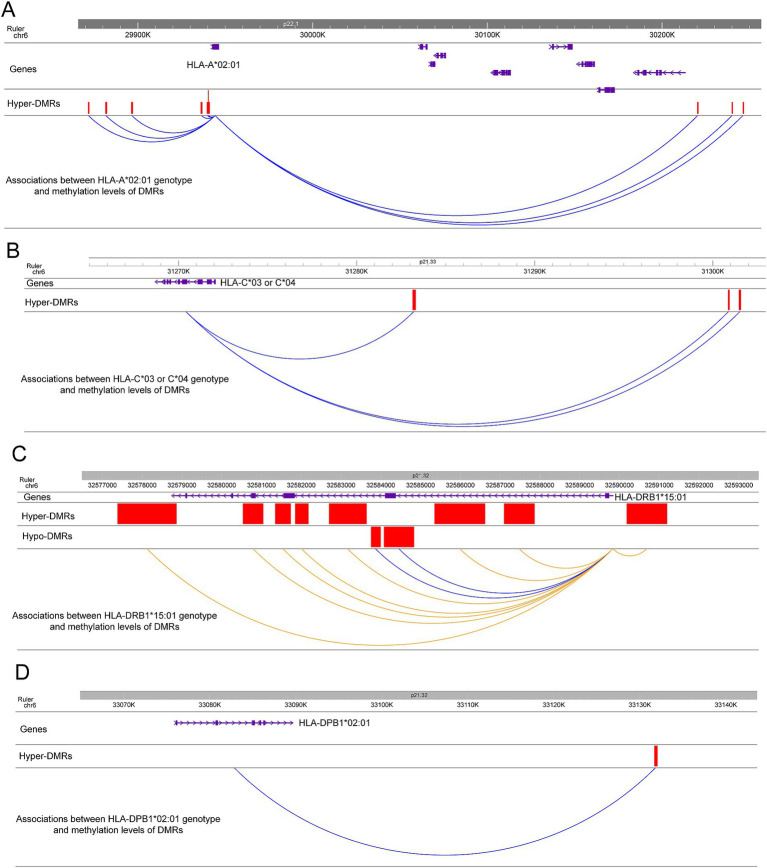
Associations between HLA genotypes and methylation levels of DMRs near or at the genes. **(A)** Associations between HLA-A*02:01 genotype and methylation levels of 9 hyper-DMRs near the gene. The correlations are colored by blue to indicate the HLA-A*02:01 carriers show lower methylation levels on the 9 hyper-DMRs compared to non-carriers. The positions of the DMRs and the gene are also depicted. **(B)** Associations between HLA-C*03 or HLA-C*04 genotype and methylation levels of 3 hyper-DMRs near the gene. The correlations are colored by blue to indicate the HLA-C*03 or HLA-C*04 carriers show lower methylation levels on the 3 hyper-DMRs compared to non-carriers. The positions of the DMRs and the gene are also depicted. **(C)** Associations between HLA-DRB1*15*01 genotype and methylation levels of 10 DMRs at the gene. Blue colored correlations indicate the HLA-DRB1*15*01 carriers show lower methylation levels on the 2 hypo-DMRs compared to non-carriers. Orange colored correlations indicate the HLA-DRB1*15*01 carriers show higher methylation levels on the 8 hyper-DMRs compared to non-carriers. The positions of the DMRs and the gene are also depicted. **(D)** Associations between HLA-DPB1*02:01 genotype and methylation levels of 1 hyper-DMR near the gene. The correlation is colored by blue to indicate the HLA-DPB1*02:01 carriers show lower methylation levels on the hyper-DMR compared to non-carriers. The positions of the DMR and the gene are also depicted.

In agreement with previous studies showing that the HLA-A*02:01 genotype has a moderately protective effect in MS (7, 17), the frequency of HLA-A*02:01 is lower in cases compared to controls (30.07% vs. 44.19%, *p* = 0.017). Interestingly, HLA-A*02:01 carriers show significantly lower methylation levels on the 9 associated hyper-DMRs compared to non-carriers (Figure 3A and Supplementary Figure S2A) with clear dose response to 0, 1, or 2 copies of the allele. Similarly, individuals with the moderately protective HLA-C*03 or HLA-C*04 genotypes (37.06% cases vs. 47.29% controls, *p* = 0.1) have significantly lower methylation levels on 3 hyper-DMRs near the gene compared to non-carriers (Figure 3B and Supplementary Figure S2B).

We next analyzed the correlations between *HLA-DRB1* genotypes and methylation levels of 10 DMRs (2 hypo-DMRs, 8 hyper-DMRs) located in the gene (Figure 3C). In this dataset, the frequency of HLA-DRB1*15:01 was significantly higher in MS participants compared to healthy controls, as expected (44.76% vs. 21.71%, *p* = 2.491e-06). HLA-DRB1*15:01 carriers showed significantly higher methylation levels on 8 hyper-DMRs (Figure 3C and Supplementary Figure S2C) and lower methylation levels on 2 hypo-DMRs compared to non-carriers (Figure 3C and Supplementary Figure S2C). Also in the class II region, the frequency of HLA-DPB1*02:01 was significantly lower in MS participants compared to healthy controls (16.79% vs. 30.95%, *p* = 0.009). When analyzing the correlation between methylation levels of DMRs and *HLA-DPB1* genotypes, we found that the individuals with the HLA-DPB1*02:01 genotype have significantly lower methylation levels on one hyper-DMR 42-kb downstream of *HLA-DPB1* gene compared to non-carriers (Figure 3D and Supplementary Figure S2D).

### mQTLs in the MHC region

**Figure 4:**
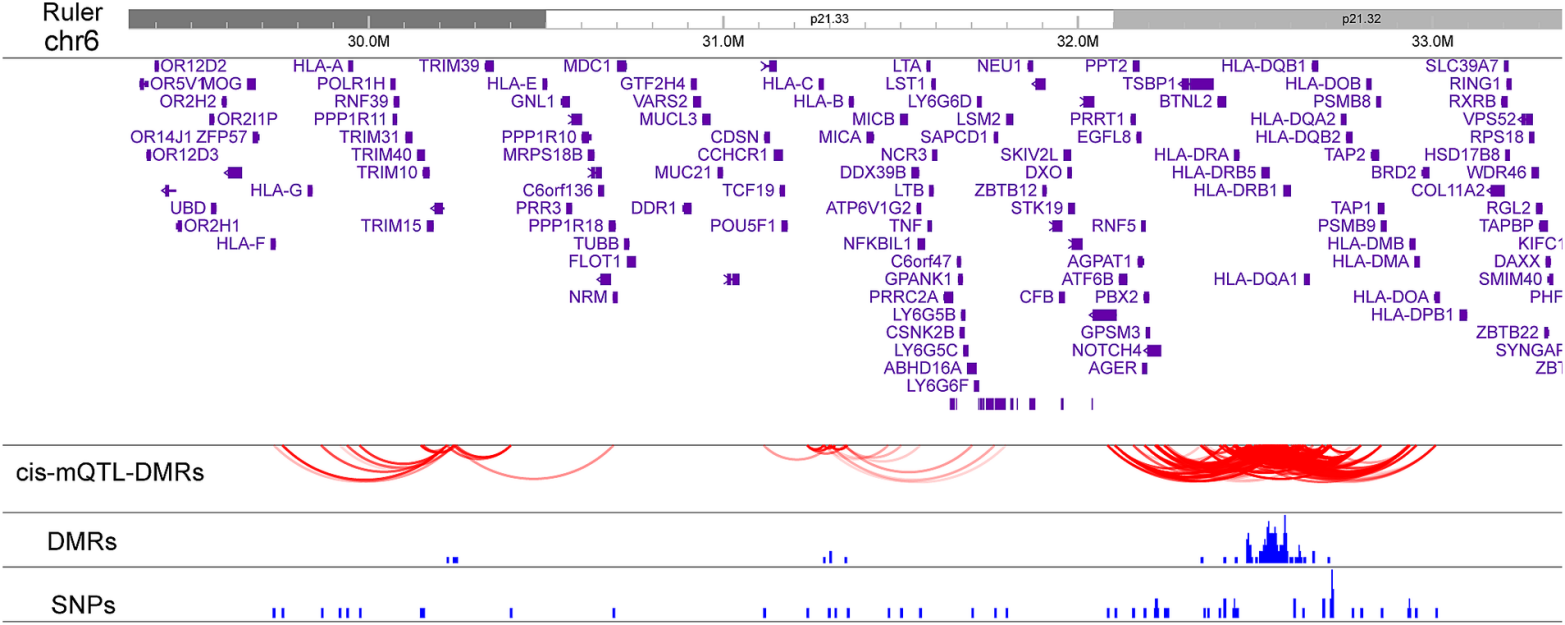
cis-mQTLs associated with MS DMR methylation levels. The plot displays paired associations between cis-mQTLs and DMRs. The positions of the DMRs and SNPs are also depicted.

Statistical associations between the DMRs methylation levels and SNPs (linkage disequilibrium *R*^2^ < 0.5) located within 500 kb up/downstream of each DMR were tested in the dataset. A total of 8,348 cis-mQTL-DMR paired associations from 485 unique SNPs and 132 DMRs were identified with FDR < 0.05. Causal inference test (CIT) analysis was performed to assess whether DNA methylation directly mediates the relationship between the genetic variants and disease phenotype. Among the 8,348 pairs, 643 cis-mQTL-DMR paired associations were significant (Supplementary Table S5). These 643 cis-mQTL-DMR paired include 55 unique SNPs and 71 unique DMRs. Notably, 46 of the 55 SNPs were associated with MS susceptibility with nominal significance (*p* < 0.05) in the recent MS GWAS study while 25 of the 55 SNPs are in linkage disequilibrium (LD) (*R*^2^ > 0.1) with 8 independent genome-wide significant associations (1) (Supplementary Table S6). In the class I region, rs2156875, a SNP in LD with MS genome-wide significant association rs2523500, behaves as a cis-mQTL affecting the DMR methylation levels in chr6: 31301483-31301548 (Supplementary Figure S3). The MHC class II region contained a high density of cis-DMR-mQTL pairs, with 34 unique SNPs and 64 unique DMRs (Figure 4). Three DMRs near *HLA-C*, *LINC02571*, and *MIR6891* genes are associated with 11 SNPs located within or near *C6orf15*, *HLA-C*, *MICB*, *LINC02571*, *HCP5*, *MIR4646*, *MIR6891*, *VARS*, *SAPCD1-AS1*, and *NFKBIL1* (Figure 4). Another three DMRs located at or near *TRIM26* and *HLA-L* are associated with 10 SNPs located within or near *HCG4B*, *HCG9*, *HCP5B*, *HLA-A*, *HLA-F*, *IFITM4P*, *NRM*, *RPP21*, *TRIM10*, and *TRIM40* (Figure 4).

## Discussion

A long-standing literature suggests that abnormal regulation and expression of *HLA* genes relates to autoimmune disease risk and phenotype including MS, conceivably operating through enhanced antigen presentation and secretion of pro-inflammatory cytokines (23–25). In the present study, base-resolution blood DNA methylation maps of the MHC generated for 147 treatment naïve MS patients and 129 healthy controls by targeted bisulfite sequencing, found significant MS-associated methylation changes that correlate with *HLA* genotypes. The effect of DNA methylation on gene expression depends on the genomic contexts: transcriptional start sites, regulatory elements, gene bodies, and repeat sequences. Nearly half the identified DMRs overlap with the retrotransposons L1 and Alu elements. Aberrant DNA methylation of interspersed repetitive sequences such as L1 and Alu have been linked to cancer, autoimmunity, and psychiatric diseases (26–29). Important gaps remain in understanding the mechanisms underlying these associations that are conceivably related to the abnormal regulation of neighboring genes. Several studies demonstrated that L1 and Alu elements can regulate the expression of *HLA* genes and other immune-related genes (30–32). Collectively, these findings support the importance of DNA methylation changes in repetitive elements driving the abnormal regulation of *HLA* in MS.

The well-established HLA-DRB1*15:01 risk genotype is associated with higher methylation levels on 8 DMRs and lower methylation levels on 2 DMRs across the gene. Notably, the hypomethylated sites map to the most variable exon 2, consistent with a recent report employing the Illumina array-based methodology showing HLA-DRB1*15:01 exon 2 hypomethylation in MS (9). The associations between HLA-DRB1*15:01 genotype and 8 hyper-DMRs have not been reported by previous studies (8, 9, 33). Classically, DNA methylation is negatively associated with gene expression. However, DNA hypermethylation can be also associated with upregulation of gene expression (34, 35). The presence of both hyper-and hypomethylated sites within a single gene, in this case *HLA-DRB1* reflects a complex, genomic context dependent cumulative regulation.

With limited coverage on the MHC region, previous array-based methods consistently reported the DMRs at *HLA-DRB1* (8, 9, 33). Here, using MHC-targeted BS-seq based method, we were also able to identify an array of DMRs at the MHC class I region and further integrate with *HLA* genetic data. *HLA-A* allelic diversity is linked to differences in levels of gene expression due in part to differential methylation patterns (36). Here we found lower methylation levels in 9 DMRs in HLA-A*02:01, an allele consistently associated with MS resistance (5, 7, 37). Interestingly, HLA-A*02:01 and HLA-DRB1*15:01 were, respectively, associated with lower and higher Epstein Barr Virus (EBV) viral load (38), broadly considered a trigger for the development of MS (39), prompting us to suggest a key role for epigenetic regulation of MHC expression in the initiation of MS. Additionally, the 3 hypermethylated sites associated with HLA-C*03 or HLA-C*04 may affect the interaction with killer cell immunoglobulin receptors (KIRs) and of natural killer (NK) cell activity (40–42). Finally, MS GWAS identified two statistically independent effects associated with the *HLA-DPB1* gene (1). We report here the higher methylation in 1 DMR is related to lower frequency of HLA-DPB1*02:01 genotype in MS, an allele associated in some studies with disease protection (43). Larger and ancestrally-diverse studies will be required to assess the DMRs associations with other *HLA* MS-relevant alleles (44–47).

Genetic variations can regulate DNA methylation patterns. We identified 485 cis-mQTLs associated with 132 DMRs. The CIT testing supported causal relationships between 55 genetic variants and MS disease status, with the majority residing in the MHC class II region, mediated by DNA methylation on 71 DMRs. In agreement with MHC region hosts the strongest MS associations, most of the mQTLs involved in the causal mediation relationships are significantly associated with MS. Our analysis shows that DNA methylation levels can mediate MS genetic risk within the MHC, especially the MHC class II region, consistent with previous studies employing the Illumina array-based methodology showing DNA methylation can mediate the genetic risk within MHC in MS (9).

In the present study we also identified 17 DMRs located in or near non-HLA genes. Among them, one DMR located telomeric of *DRB1*, in intron two of NF-kB inhibitor like 1 (*NFKBIL1*), a nominated susceptibility gene for MS (1). *NFKBIL1* is involved in mRNA processing (48, 49), and the alternative splicing of both human immune-related and viral genes (48). The observed hypermethylation of *NFKB1L1* in the MS genome and HLA-independent susceptibility effects (1) provide a steppingstone for mechanistic studies of a key component of the inflammatory response. The understanding of how epigenetic regulation is functionally linked to MS initiation and progression may provide novel diagnostic, prognostic, and intervention opportunities.

## Data availability statement

The datasets presented in this study can be found in online repositories. The name of the repository and accession number can be found at: National Center for Biotechnology Information (NCBI) Gene Expression Omnibus (GEO), https://www.ncbi.nlm.nih.gov/geo/, GSE235106.

## Ethics statement

The studies involving humans were approved by UCSF Institutional Review Board. The studies were conducted in accordance with the local legislation and institutional requirements. The participants provided their written informed consent to participate in this study.

## Author contributions

QM: Conceptualization, Data curation, Formal analysis, Funding acquisition, Methodology, Supervision, Visualization, Writing – original draft, Writing – review & editing. DA: Methodology, Writing – review & editing. GM-M: Data curation, Methodology, Writing – review & editing. SC: Methodology, Writing – review & editing. KO: Methodology, Writing – review & editing. BC: Resources, Writing – review & editing. SH: Funding acquisition, Resources, Writing – review & editing. AD: Methodology, Writing – review & editing. JH: Methodology, Writing – review & editing, Funding acquisition. PN: Methodology, Writing – review & editing. MF-V: Data curation, Writing – review & editing. JO: Conceptualization, Funding acquisition, Supervision, Writing – original draft, Writing – review & editing.
