# Supplementary material for: High-resolution DNA methylation screening of the major histocompatibility complex in multiple sclerosis
Source: Front Neurol. 2023 Dec 8;14:1326738. doi: 10.3389/fneur.2023.1326738 (PMC10739394; doi:10.3389/fneur.2023.1326738)
Supplement: Supplementary file 1 [file Data_Sheet_1.pdf]

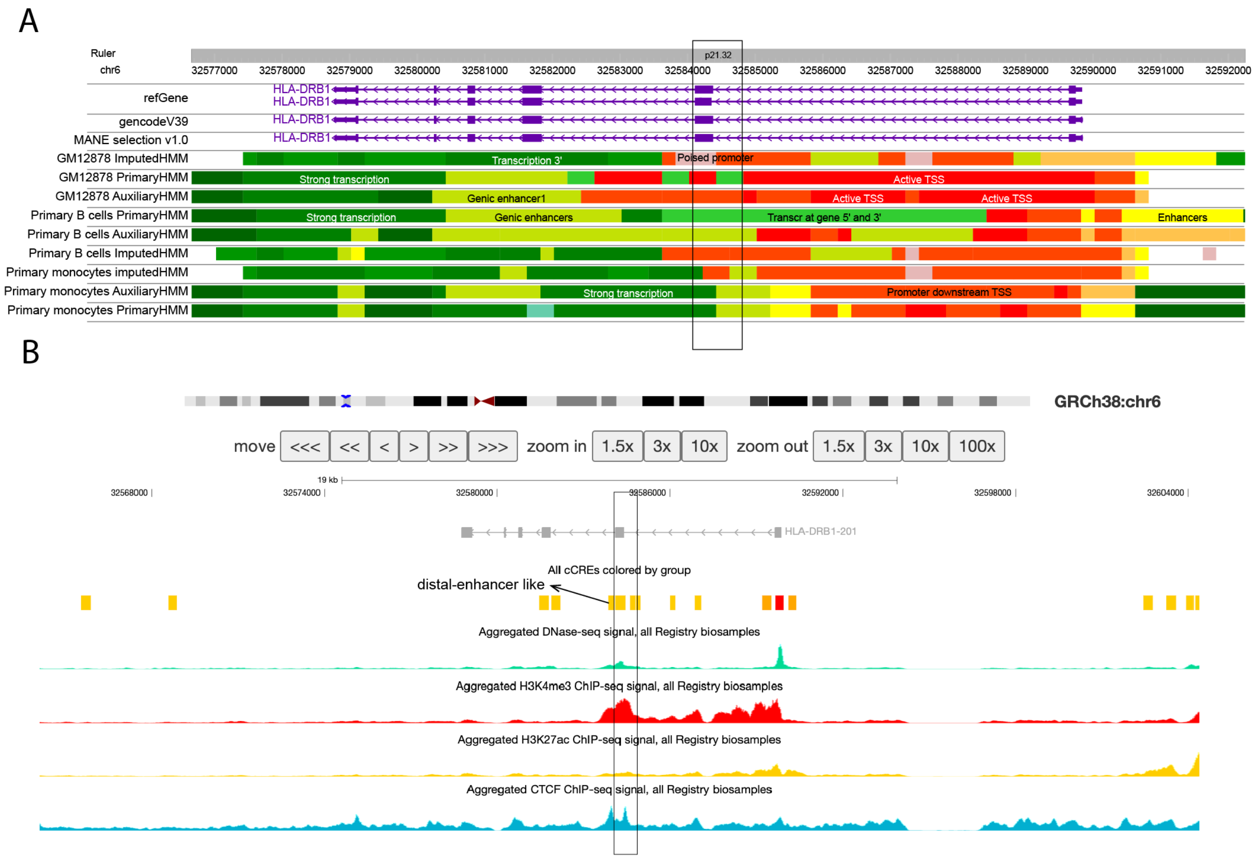

Figure S1. The hypo-DMR in the HLA-DRB1 is predicted as poised/active promoter and distal enhancer. (**A**) WashU Epigenome Browser snapshot displaying the chromatin state segmentation of HLA-DRB1 gene for B cells and monocytes based on ChromHMM from Roadmap. (**B**) The genome browser view displaying the candidate cis-Regulatory Elements (cCREs) in HLA-DRB1 gene derived from ENCODE data. cCREs with distal enhancer-like signatures have high DNase and H3K27ac max-Z scores and is located more than 2 kb of a transcription start site (TSS). The hypo-DMR is depicted with black box.

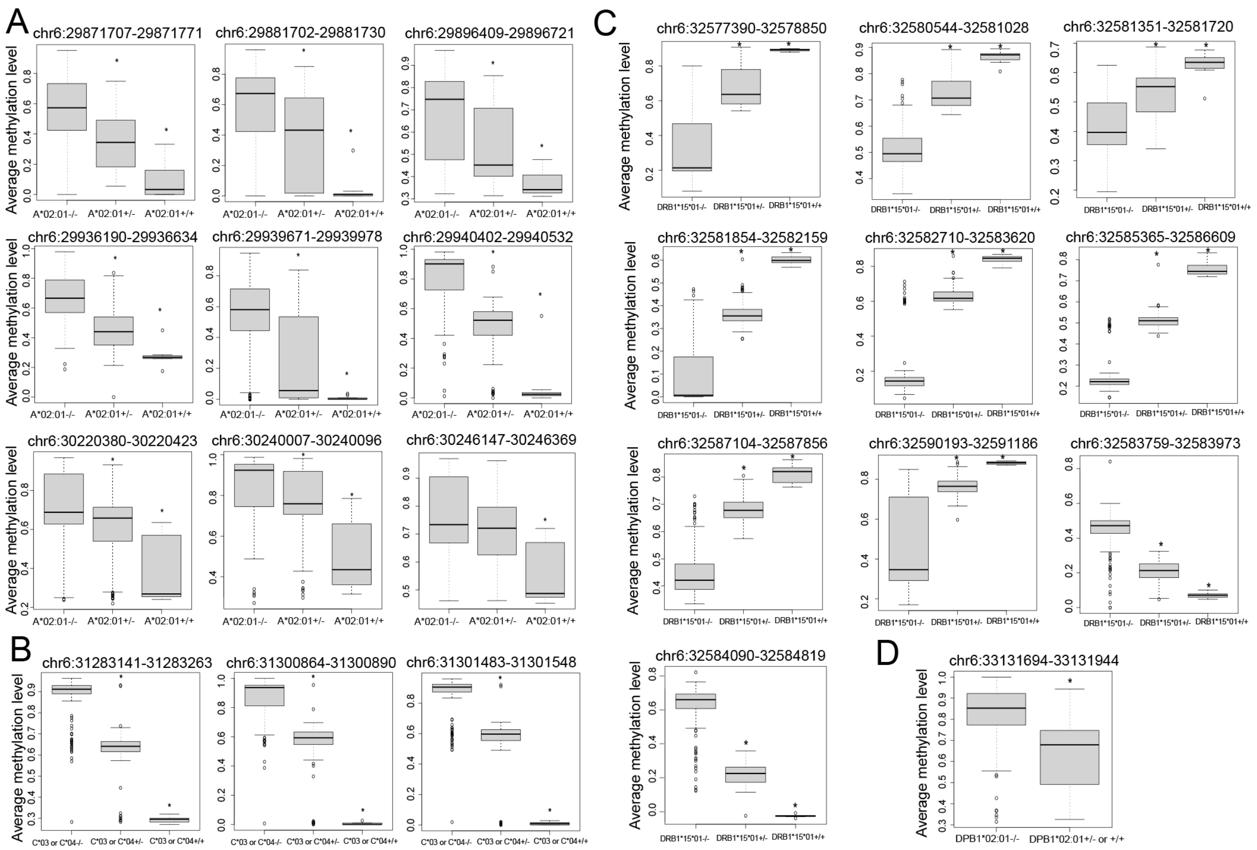

Figure S2. HLA genotypes are significantly associated with lower or higher methylation levels of the DMRs. (**A**) Boxplots represent average methylation levels on 9 hyper-DMRs according to HLA-A*02:01 genotype: non-carriers (−/−), heterozygous (+/−), homozygous (+/+). * P<0.05. (**B**) Boxplots represent average methylation levels on 3 hyper-DMRs according to HLA-C*03 or HLA-C*04 genotype: non-carriers (−/−), heterozygous (+/−), homozygous (+/+). * P<0.05. (**C**) Boxplot represents average methylation levels on 8 hyper-DMRs and 2 hypo-DMRs according to HLA-DRB1*15*01 genotype: non-carriers (−/−), heterozygous (+/−), homozygous (+/+). * P<0.05. (**D**) Boxplot represents average methylation levels on 1 hyper-DMR according to HLA-DPB1*02:01 genotype: non-carriers (−/−), heterozygous (+/−), homozygous (+/+). * P<0.05.

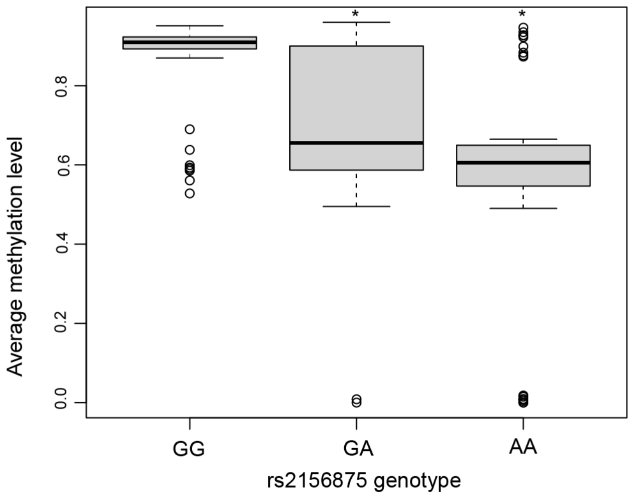

Figure S3. SNP rs2156875 is a cis-mQTL associated with average methylation levels of the DMR at genetic positions 31301483-31301548 (hg38) on chromosome 6. Boxplot represents average methylation levels on the DMR according to rs2156875 genotypes: GG, GA, AA. * P<0.05.

Table S1. DMRs in multiple sclerosis.

Table S2. The MS risk variants with 100k around regions overlapped with DMRs within MHC.

Table S3. MS-associated DMRs that overlapped with repetitive elements.

Table S4. The significance of the correlations (P-value) between HLA genotypes and methylation levels of DMRs.

Table S5. The significant cis-mQTL-DMR paired associations.

Table S6. The cis-mQTLs that are in linkage disequilibrium (LD) with independent genome-wide significant associations within MHC region.
